# Optimal Short-Time Acquisition Schemes in High Angular Resolution Diffusion-Weighted Imaging

**DOI:** 10.1155/2013/658583

**Published:** 2013-03-11

**Authors:** V. Prčkovska, H. C. Achterberg, M. Bastiani, P. Pullens, E. Balmashnova, B. M. ter Haar Romeny, A. Vilanova, A. Roebroeck

**Affiliations:** ^1^Department of BioMedical Engineering, Biomedical Image Analysis & Interpretation, Eindhoven University of Technology, Eindhoven, The Netherlands; ^2^Faculty of Psychology & Neuroscience, Maastricht Brain Imaging Center, Maastricht University, Maastricht, The Netherlands; ^3^Biomedical MR Imaging and Spectroscopy Group, Image Sciences Institute, University Medical Centre Utrecht, Bolognalaan 50, 3584 CJ Utrecht, The Netherlands; ^4^Department of Mathematics and Computer Science, Eindhoven University of Technology, Eindhoven, The Netherlands

## Abstract

This work investigates the possibilities of applying high-angular-resolution-diffusion-imaging- (HARDI-) based methods in a clinical setting by investigating the performance of non-Gaussian diffusion probability density function (PDF) estimation for a range of *b*-values and diffusion gradient direction tables. It does so at realistic SNR levels achievable in limited time on a high-performance 3T system for the whole human brain *in vivo*. We use both computational simulations and *in vivo* brain scans to quantify the angular resolution of two selected reconstruction methods: Q-ball imaging and the diffusion orientation transform. We propose a new analytical solution to the ODF derived from the DOT. Both techniques are analytical decomposition approaches that require identical acquisition and modest postprocessing times and, given the proposed modifications of the DOT, can be analyzed in a similar fashion. We find that an optimal HARDI protocol given a stringent time constraint (<10 min) combines a moderate *b*-value (around 2000 s/mm^2^) with a relatively low number of acquired directions (>48). Our findings generalize to other methods and additional improvements in MR acquisition techniques.

## 1. Introduction

Diffusion-weighted magnetic resonance imaging (DW-MRI) is a clinical medical imaging technique that provides a unique view on the structure of brain white matter *in vivo*. Right from its early stages, in the early 1990s, DW-MRI was perceived to have immediate value for the evaluation of neuropathologies such as acute ischemic stroke. Since then, numerous advents in diffusion imaging technology have greatly augmented image quality unraveling new clinical applications. Moreover, the debut of diffusion tensor imaging (DTI) and fiber tractography enabled a completely new, noninvasive view on white matter fibre bundles connecting gray matter neural populations, of increasing importance for cognitive neuroimaging applications. With DTI and fiber tractography, the understanding of several neurological and psychiatric disorders, such as schizophrenia, traumas, stroke, and edemas, has been increased, and they have also been applied clinically to aid presurgical planning before intracranial mass resections.

In DW-MRI, white matter fiber bundles are probed indirectly by measuring the directional specificity (anisotropy) of local water diffusion. During a small time interval (the “so-called” effective diffusion time *t*), at each location of the tissue, the diffusion causes a displacement of water molecules. Postprocessing of diffusion-weighted images is fundamentally aimed at calculating the probability density function (PDF) *p*(**r**), for this displacement of water molecules in each imaging voxel, where **r** is the 3D displacement vector. Since fiber bundles in tissue allow relatively free diffusion of water along the fibers but tend to obstruct the diffusion perpendicular to the fibers, one assumes that the reconstructed PDF, also known as the diffusion propagator, reflects the local fiber structure within a voxel. Diffusion tensor imaging (DTI) approximates the diffusion propagator by a 3D Gaussian function, which allows a relatively simple estimation of a small number of sampled diffusion directions [1]. However, it has recently become clear that a simple unimodal Gaussian approximation of the diffusion PDF cannot represent interesting complex fiber architecture such as single fiber bundles curving or fanning or multiple fiber bundles with different directions contained in a single imaging voxel [2–6]. Consequently, much of the modeling efforts have aimed at estimating multimodal (non-Gaussian) characterizations of the diffusion PDF that capture complex fiber architecture, particularly the presence of multiple fiber populations in single voxels. Although the full diffusion PDF provides the most extensive information about the structure of the material at microscopic scale, for many applications like fiber tracking, it is the orientation that is of most importance. Therefore, a simplified function is often derived, called the orientation distribution function (ODF), defined as the radial projection of the diffusion function. Crucially, a characterization of the ODF generally puts much lower requirements on the sampling of diffusion directions and, particularly, degrees of diffusion weighting than a characterization of the full diffusion PDF. Many recent modeling techniques, such as Q-ball [7–9], the DOT [10], and spherical deconvolution (SD) [11], focus on obtaining a low-variance unbiased estimate of the diffusion ODF (or the “fiber” ODF that represents probabilities of fiber orientations, rather than water diffusion positions) with only moderate acquisition requirements. The required acquisition is generally a high angular resolution diffusion imaging (HARDI) acquisition that acquires a large number of diffusion directions at a single level of diffusion weighting (a “moderate” *q*-space sampling on a single spherical shell).

The general convention for conducting HARDI acquisitions in presenting, extending, or testing non-Gaussian techniques is to sample as many gradient direction vectors, at as high as possible *b*-values that the scanner and the subject stamina allow, while maintaining sufficiently high SNR. Tuch [4] reports *b*-values of 12000 s/mm^2^ and number of gradients (NG) of about 492, whereas Hagmann et al. [12] reports *b* > 4000 s/mm^2^ and NG > 60, and Descoteaux [13] *b* > 1000 s/mm^2^ (although he states that *b* > 3000 s/mm^2^ is desirable) and NG > 60. Compared to the modest *q*-space sampling for DTI (with total acquisition time of 3–6 minutes [12]), the HARDI techniques have 3-4 times longer acquisition times on average. Although there is some variation in the requirements between different techniques, generally a large number of diffusion directions (on the order of 50–300) at a considerable level of diffusion weighting (on the order of *b* = 1000–4000 s/mm^2^) with a high signal-to-noise ratio is needed. In addition, fiber tractography requires whole-brain coverage at a high spatial resolution (i.e., small isotropic voxel size). However, each of these requirements generally increase the acquisition time needed, potentially making HARDI unattractive for clinical applications or cognitive neuroscience investigations that want to add a limited time diffusion protocol to their fMRI measurements.

Therefore, an important combination of open questions about the measurement requirements of these HARDI modeling techniques arises. To what degree does the dense angular gradient sampling improve the accuracy of these techniques, particularly in less favorable SNR regimes? Do high *b*-values contribute to the accuracy of the reconstructions, given the SNR penalty that needs to be payed? More generally, given the multiway trade-off between spatial resolution, angular resolution, *b*-value, SNR, and measurement time, what are suitable settings for these parameters in a HARDI protocol for a modern 3T scanner and considerable time constraints (10–20 minutes) typical for a clinical or fMRI experiment setting? Finding the optimal parameters for the acquisition schemes in terms of both the direction sets and *b*-value has been an open issue in HARDI acquisitions and a few investigations have partially tackled this issue. Crucially, all of these studies focussed on a situation where acquisition time constraints were very lenient (long diffusion-only protocols on experienced subjects) or absent (phantom and simulation studies). This allowed them to perform their investigation of optimal sampling schemes in a very favorable signal-to-noise ratio (SNR) regime.

This work investigates the possibilities of applying HARDI-based methods in a clinical setting by investigating the performance of non-Gaussian diffusion PDF estimation for a range of *b*-values and diffusion gradient direction tables. It does so at realistic SNR levels achievable in limited time on a high performance 3T system for the whole human brain *in vivo*. We use both computational simulations and *in vivo* brain scans to quantify the angular resolution of two selected reconstruction methods: Q-ball imaging and the diffusion orientation transform (DOT) (and its derivations). Since the output of the DOT is the diffusion PDF, for fair comparison in this work, we derive the ODFs from the DOT and quantify the angular resolution in a similar fashion as for Q-ball. Moreover, we propose an analytical solution of the ODF derived from the DOT. Both techniques are analytical decomposition approaches that require identical acquisition and modest postprocessing times, and given the modifications of the DOT can be analyzed in a similar fashion. Our findings generalize to other methods and additional improvements in MR acquisition techniques.

## 2. Methods

### 2.1. Q-Ball Imaging

In DW-MRI, the obtained measurements do not give the diffusion propagator or its derivations, such as the ODF, directly. We review the transformation from measured signals to reconstructed directional profiles under a few of the most used modeling assumptions and conditions.

The simplest transformation that relates the measured signal *S*(**q**) and the full PDF is the 3D Fourier transform as shown by Callaghan et al. [14]:

(1) $$\begin{array}{c} p\left(r\right)=𝔉\left[E\left(q\right)\right], \end{array}$$

where *E*(**q**) = *S*(**q**)/*S*_0_ is the normalized signal, *S*_0_ is the unweighted or zero-weighted baseline signal obtained without any applied diffusion gradients, and **q** is the diffusion 3D wavevector that characterizes the direction and strength of the diffusion weighting obtained from the nuclear spins in the presence of a diffusion sensitizing magnetic gradient. This relationship has been exploited in diffusion spectrum imaging (DSI) [15, 16], where the full PDF is reconstructed by a discrete Fourier transformation of a densely sampled (on a cartesian grid) signal *S*(**q**). However, the requirements on measurement duration in DSI are considerable, and one might not be always interested in reconstructing the full and exact PDF. Therefore, depending on the application, simpler and less accurate methods are usually applied. For the purpose of simplification, many assumptions about the signal and/or the underlying PDF can be made, hence many different modeling techniques have been proposed in the literature among which are the follwing.

(1) *Assumption of a mono-exponential signal decay and Gaussian PDF:* the simplest approach is DTI. In DTI one assumes a Gaussian diffusion propagator which results via Fourier transformation in a Gaussian signal *S*(**q**) = *S*_0_ *e* ^−*bD*(**g**)^, where *b* = |**q**|^2^ *t* is the acquisition parameter, *t* is the effective diffusion time, and **g** = **q**/|**q**|. The apparent diffusivity *D*(**g**) is modeled by a second order tensor **D**, such that with gradient **g** one assumes *D*(**g**) = **g** ^T^ · **D** · **g**. DTI requires very modest gradient **g** samplings on a *q*-shell [15] to determine its coefficients. However, this approach is limited, as it can resolve only one fiber bundle per voxel.
(2) *Assumption of a monoexponential signal decay S*(**q**) = *S*_0_ *e* ^−*bD*(**g**)^ *:* in HARDI, to increase the angular resolution, the apparent diffusivity *D*(**g**) is modeled in a less crude way by spherical harmonics (SH) or high order tensors (HOT) [3, 6, 17]. This enhances the characterization of tissue properties, but also increases the number of necessary *S*(**q**) samples, and gives incorrect results regarding the directions of the fiber crossings as shown by Özarslan et al. [10]. DOT maps the ADC into a probability function *p*(**r**) by solving the Fourier transform using the Rayleigh expansion of a plane wave in spherical coordinates. It is worth mentioning that DOT permits multi-exponential signal decay which allows the exploration of multiple *q*-shell samplings.
(3) *No assumption about signal decay or the underlying diffusion process:* Q-ball imaging [4] simply maps the measurements on a *q*-shell via the Funk-Radon transform into another function on a sphere called orientation distribution function *ψ*(**u**) (ODF). This ODF *ψ*(**u**) is the integral in radial direction of the true diffusion propagator *p*(**r**):
  (2) $$\begin{array}{c} \psi \left(u\right)=\int_{0}^{\infty }p\left(r,u\right)dr, \end{array}$$
  where **u** = **r**/|**r**|. One of the biggest recently developed advantages of Q-ball is its analytical solution of the Funk-Radon transform [8, 9, 18] utilizing a basis of spherical harmonics (SH), which significantly improves its performance.
(4) Lately, *spherical deconvolution (SD) methods* proposed in the literature [11, 19] are becoming popular, especially for fiber tracking purposes due to sharper profiles of the reconstructed fiber orientation distribution (FOD) *F*. One assumes, that the DWI signal *S*(**q**) can be modeled by a superposition of fiber bundle signals *R* via the convolution *S* = *F*∗*R*. The FOD that one obtains by a spherical deconvolution of *S* with *R* reflects the angular distribution of fiber bundles. One has to keep in mind that this approach incorporates assumptions about the single fiber response *R*. Recently, it has been shown that some of the bias in the fiber extraction from ODF and FOD (especially under small angles of crossings) can be improved using tensor decomposition approach as in the work of Schultz and Seidel [20].

There are many other diffusion modeling techniques in the literature, but these exceed the scope of this paper. In our analysis we will include the analytical Q-ball proposed by Descoteaux et al. [8], since it is an analytical solution to the numerical ODF [15], and it has advantages as such. In our comparison, we excluded the Laplace-Beltrami regularization proposed by Descoteaux et al. [8], due to the increase of the complexity of the parameter space examined in this work as well as the difficulties that arise when trying to compare this method to the DOT (see below) that is proposed without any regularization. However, due to the assumption of the monoexponential decay for the signal attenuation in DOT, a smoothing in *q*-space can be assumed and has advantages for smaller *b*-values where the monoexponential signal decay holds.

### 2.2. Diffusion Orientation Transform

The DOT [10] reconstructs the diffusion propagator *p*(*R*_0_ **u**) at a given radius *R*_0_ and a unit vector **u**. In the DOT, the radius for reconstructing a single shell of the PDF *R*_0_ has to be determined heuristically, and this process is not a trivial task, especially in the case of real data. *R*_0_ is highly dependent on the scanning parameters, especially the *b*-value, and possibly the tissue properties of the voxel that we try to resolve.

#### 2.2.1. Numerical DOT-ODF

**Figure 1:**
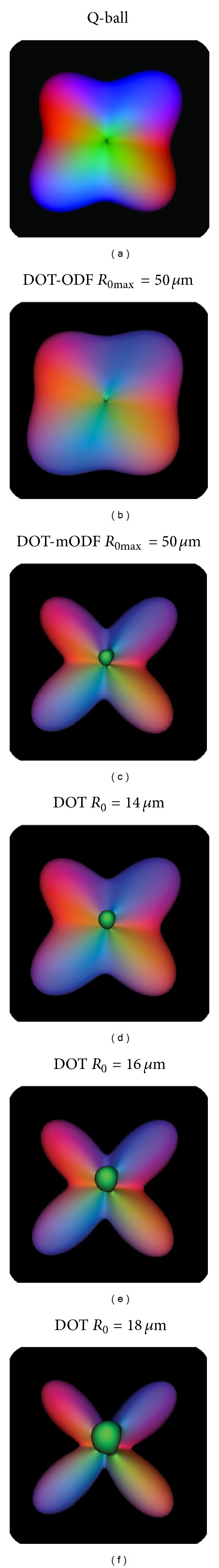
Profiles of the DOT and the proposed derivations in a single crossing voxel from a real brain in the area of the centrum semiovale. The data is acquired at b = 1500 s/mm^2^ and 60 gradient directions. For the DOT, the effective diffusion time t is set to 25 ms. We observe that the DOT-mODF performs similarly as the DOT reconstructed at R_0_ = 16 *μ*m. Additionally, Q-ball and the DOT-ODF show similar diffusion profiles.

To avoid the selection of *R*_0_ and inspired by definitions of the ODF from *q*-ball imaging [4] and the marginal ODF (mODF) from diffusion spectrum imaging (DSI) [21], we propose similar ODFs computed from the DOT as

(3) $$\begin{array}{c} \psi_{\text{DOT-ODF}}\left(\theta ,\phi \right)=\int_{0}^{\infty }p\left(R_{0},\theta ,\phi \right)dR_{0},\\ \;\;\psi_{\text{DOT-mODF}}\left(\theta ,\phi \right)=\int_{0}^{\infty }p\left(R_{0},\theta ,\phi \right)R_{0}^{2}dR_{0}, \end{array}$$

where *p*(*R*_0_, *θ*, *ϕ*) = *p*(*R*_0_ **u**) is the PDF computed from the DOT [10], with **u** as a unit vector defined by (*θ*, *ϕ*), where *θ* ∈ [0, *π*], *ϕ* ∈ [0,2*π*]. In our calculations we truncate the integrals (summations in a discrete form) to an arbitrary high value, *R*_0max⁡_, and then perform a large number of summations from the full diffusion PDF. In Figure 1, we illustrate the diffusion profiles of the DOT and the proposed derivations. We observe that the DOT-mODF performs similarly as the DOT itself, and the Q-ball and DOT-ODF profiles exhibit resemblance as well.

#### 2.2.2. Analytical DOT-ODF

Even though the sensitivity on the parameter *R*_0max⁡_ for the numerical DOT-ODF is less than for the original formulation, an arbitrary high value for *R*_0max⁡_ still must be specified. Additionally, a high number of integrations (or in discrete sense summations) have to be performed in each orientation of the full PDF to calculate the ODF value in that direction. Therefore, we propose the analytical derivation of the DOT-ODF explained in detail in Appendix A. The advantage of using the analytical DOT-ODF, is not only in reducing the computational expenses but also in avoiding the *R*_0_ selection problem entirely. This way, we propose an ODF that can be easily compared to the ODFs proposed by Tuch [15] and later Descoteaux et al. [8]. Moreover, the analytical DOT-ODF method is less computationally expensive than the numerical method, since it does not require sampling of multiple shells of the PDF for summations. The required computational time is the same as for the analytical Q-ball.

### 2.3. Data

Previous studies have largely focussed on either software simulations or real MRI data. Where the analysis and validation of the simulated data can be absolutely quantitative, one must be cautious in generalizing results since simulated properties of tissue, acquisition, and noise parameters could be unrealistic for any simulation. In contradistinction, where full realism is evidently obtained in actual *in vivo* data, the analysis must be relatively qualitative because of the absence of an exact gold standard. To quantify the accuracy of the selected modeling techniques, we generate both synthetic data of two fiber crossings, and we extend our analysis to real data acquired *in vivo* on a human subject. In this way, we combine the advantage of a ground truth and full knowledge and control of all parameters in the synthetic data simulations, with the guaranteed realism of the tissue, acquisition, and noise parameters in the real data.

#### 2.3.1. Synthetic Data

We generate synthetic software data according to the Söderman and Jönsson's [22] model used in Özarslan et al. [10], Brampoutis et al. [23], and von dem Hagen and Henkelman [24].

We fix the parameters for this model similarly to Özarslan et al. [10] to fiber length *L* = 5 mm, fiber radius *ρ* = 5 *μ*m, and free diffusion coefficient *D*_0_ = 2.02 × 10^−3^ mm^2^/s.

We calculate the effective diffusion time as *t* = Δ − *δ*/3 where the *δ*/3 correction is due to the diffusion which occurs during the time in which the gradients are on.

Using Söderman's model we create two fiber crossings under angles of: 40°, 45°, 50°, 55°, 60°, and 90°. The choice for these angles was made given the expected accuracy for Q-ball imaging [8, 25] of around 50° (however, this is strongly dependent on the *b*-value and applied regularization). To create different datasets with different acquisition parameters we vary the number of gradients and *b*-values as in Tables 1 and 2, respectively.

Additional parameters that need to be specified for the Söderman's model are the values for the gradient pulse duration *δ* and gradient spacing Δ. We use the same values as from our real acquisition protocol where we optimize the echo time TE per *b*-value.

This way we optimize the SNR per *b*-value, and the resulting profiles will be less deteriorated from noise. However, one has to be careful here because the representations of the PDFs are for different measurement times. For all the data in this work, the same parameters listed in Table 2 are used in order to allow for a fair relation between the synthetic and *in vivo* data. To create noisy synthetic data we add Gaussian noise to the real and complex part of the signal, and we vary the standard deviation depending on the analysis we want to perform.

#### 2.3.2. *In Vivo* Human Data

DW-MRI acquisitions were performed on a female subject using a twice refocused spin-echo, echo-planar imaging sequence on a Siemens Allegra 3T scanner (Siemens, Erlangen, Germany) equipped with a 40 mT/m head gradient set, an 8-channel cylindrical phased-array RF-coil. Informed consent was obtained prior to the measurement. Twenty axial slices (FOV[220 × 220] mm^2^, matrix [88 × 88][2.5 × 2.5 × 2.5] mm^3^ voxel size) were positioned through the body of the *corpus callosum* and the *centrum semiovale*. EPI acquisition was performed with 3/4 partial Fourier and a readout bandwidth of 2840 Hz/Px. Datasets were acquired with diffusion gradient sampling schemes of 24, 36, 48, 60, and 72 unique directions obtained by a static repulsion algorithm, with the diffusion-weighted volumes interleaved with zero-weighted volumes every 12th scanned diffusion gradient direction. For each gradient scheme the scans were performed with *b*-values of 1000, 1500, 2000, and 3000 s/mm^2^ (TE, resp., 77, 85, 91, and 100 ms, TR = 4000 ms throughout), using the same gradient pulse duration *δ* and gradient spacing Δ as given in Table 2. No averaging of repeated acquisition was performed, that is, there was only a single acquisition for each parameter set.

### 2.4. Analysis of Synthetic Data

To analyze the accuracy of the reconstructed HARDI models in the synthetic data sets, we report the detected angular error. We use a simple scheme for determining the angular error, that is, we find the local maxima of the reconstructed profiles on a mesh generated by tessellating icosahedron of certain order and then report the angular difference between these maxima and the simulated (true) fiber directions. To reduce the error that the discrete mesh imposes, in our analysis we use the 7th order of tessellation of the icosahedron, that imposes 1° spacing between tessellation points, thus, a maximum distance between any point and the nearest tessellation point of 0.5°. Depending on the orientation of the fibers in the space of the gradients, different errors might appear for the same simulated angle. For example, if we simulate an angle of 60° by two crossing fibers and then rotate the fibers in 3D space, one would assume that always the same angle of crossing is detected. However, in Söderman's model, different angular errors are calculated for different orientations of the fibers. In general, these errors cause less than 5° angular error, and therefore while choosing our optimal parameters for certain angular configuration, we take these errors into account. For example, for an angle of 60°, the *b*-value of 3000 s/mm^2^ might give an angular error of 4° and the *b*-value of 4000 s/mm^2^ an angular error of 2.5°. However, due to the errors imposed by the tessellation of the mesh and the orientation of the fibers as well as our choice of tolerance for 5°, we report the b-value of 3000 s/mm^2^ as preferable, since both *b*-values are within our range of tolerance, and we always report the smallest *b*-value as optimal.

### 2.5. Analysis of Human Data

#### 2.5.1. Criteria for *In Vivo* Data Analysis

**Figure 2:**
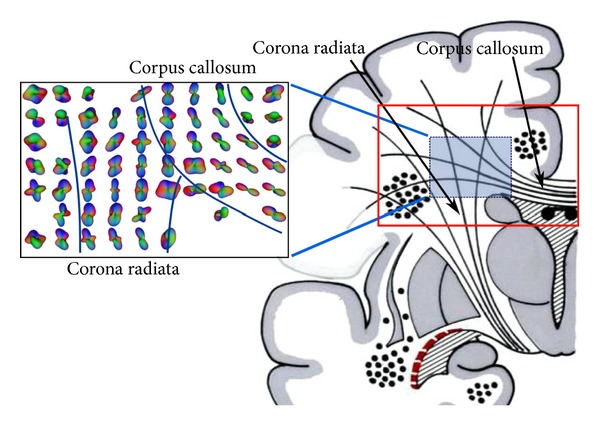
ROI used to illustrate the real data findings.

For qualitative analysis of the real data, we select the *centrum semiovale* (CS) (see Figure 2) where crossings are to be expected. This is a challenging region for DW-MRI analysis techniques, since fibers of the *corpus callosum* (CC), *corona radiata* (CR), and *superior longitudinal fasciculus* (SLF) form a three-fold crossing. A region-of-interest (ROI) was defined on a coronal slice. Red rectangle indicates the position of the slices in the acquisition.

The qualitative criteria for comparison are the sharpness of the profiles in CC and lower parts of the CR, the presence of two ODF maxima for the CC and CR and the spread of the their crossing in the *centrum semiovale*, and the presence of the *superior longitudinal fasciculus* in the upper corners of the region. Furthermore, the presence of spurious peaks is an indication of poor SNR and unreliably detected crossings. 

#### 2.5.2. SNR in *In Vivo* Data

We calculate the SNR in the images via the “difference method” as described by Reeder, et al. [26]. For a set of two unweighted volumes, the SNR is computed via the difference method as

(4) $$\begin{array}{c} \text{SNR}{|}_{\text{ROI}}=\frac{{\mu \left(B_{0}+B_{0}^{\prime }\right)|}_{\text{ROI}}}{{\sqrt{2}\sigma \left(B_{0}-B_{0}^{\prime }\right)|}_{\text{ROI}}}, \end{array}$$

where *B*_0_ and *B*_0_′ are two unweighted images and *μ* and *σ* are the mean and the standard deviation respectively. The SNRs, computed in an ROI of 60 voxels in the middle of the *corpus callosum,* is 23.58, 20.75, 19.29, and 18.61 at *b*-values of 1000, 1500, 2000, and 3000 s/mm^2^, respectively. The final reported SNR is calculated as the median of six SNRs, each computed from two unweighted images closest in time. Since unweighted volumes were interspersed between 12 diffusion weighted volumes, the SNRs are probably slightly under-estimated due to bulk motion and drift over the measurement time. Note that, due to the characteristics of the phased-array RF coil, the SNR in the CC in the middle of the head is a lower bound for the SNR in the rest of the scanned volume. SNR is expected to increase (quadratically) towards the periphery. 

#### 2.5.3. Scanning Time

To give an indication for the time requirements for the individual acquisition schemes, we estimate the total scanning time needed to obtain a full brain acquisition at the given parameters. Assuming at least 60 axial 2.5 mm slices would be needed for whole brain coverage and the shortest achievable TR would be used, whole brain measurement times are given in Table 3.

## 3. Results

In this section we will discuss the quantitative results from our synthetic data and give qualitative implications for the real data scanned under the same parameters. 

### 3.1. Synthetic Data Analysis

To get an indication on the optimal parameters for each simulated angle, we run a series of tests, where we determine the minimal angular error that can be obtained with the minimal combination of *b*-value and *ℓ*-order, under different gradient sampling schemes. The results are presented in Table 4.

**Figure 3:**
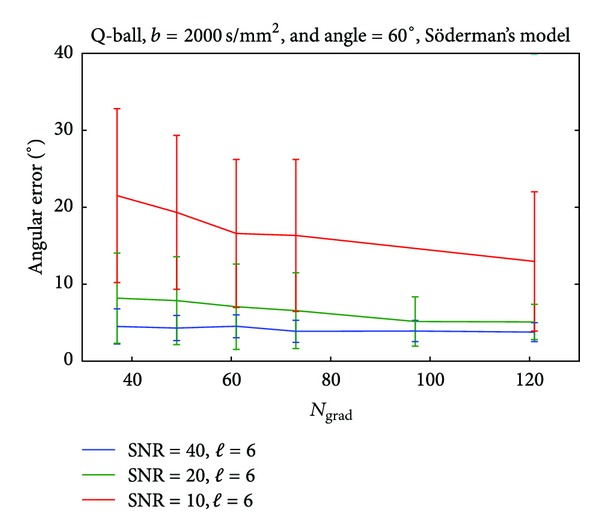
Influence of the gradient sampling schemes on the accuracy of Q-ball imaging for angle of 60°.

We observe similar trends for both of the reconstruction techniques. Both Q-ball and DOT-ODF can accurately represent crossings of 90° for *b*-values as low as 1000 s/mm^2^ and order of SH *ℓ* = 4 regardless of the choice for the gradient sampling scheme. The scenario changes for the angle of 60°, where the best choice for *b*-value recommends 3000 s/mm^2^ and higher order of SH of *ℓ* = 6. Trying to resolve smaller angles of crossings, with these analytical decomposition methods results in higher choices for *b*-values and SH order even in noiseless cases. The SNRs in clinical scanners at high *b*-values such as 4000 s/mm^2^ drop and therefore these *b*-values are not recommended. The noise corrupts the reconstructed profiles, and modeling with high orders of SH will result in capturing the noise, which is dominant in these cases. Therefore, from the noise tables (see Supplementary Material available online at http://dx.doi.org/10.1155/2013/658583) we already get the indication that the lowest angle which can be captured within reasonably imposed angular error would be around 60°. To reduce the parameter space, we therefore select this interesting angle and SH order of *ℓ* = 6 (as Table 4 indicates for this angle) and look at some interesting influences of the *b*-values and number of gradients, under different SNRs. Another motivation for this choice of reconstruction angle is the region that we select for illustrating the *in vivo* results. The *centrum semiovale* contains a large amount of voxels where the crossing angles are approximately between 60° and 90°. To investigate the effect of the gradient sampling schemes, we performed tests where for *b*-values of 2000 s/mm^2^, we vary the number of gradients under different levels of added Rician noise. In Figure 3, we report the calculated angular error and the standard deviation of 100 noise realizations for simulations of 60° angle of crossing. We observe that for SNR of about 20 (similar to our case) of the recovered angle suffers from an angular error of less than 10°. For higher SNRs (20–40), we observe that around a number of 70 gradients the graph stabilizes and we do not gain much accuracy by having denser gradient sampling schemes. The results are similar to the ones from the DOT-ODF and for completeness are reported in Figures 8, 9 and 10.

**Figure 4:**
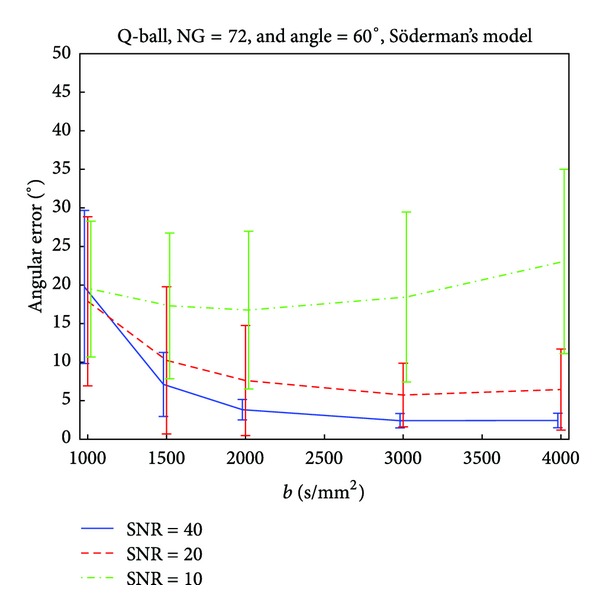
Influence of the *b*-value on the accuracy of Q-ball imaging for a crossing angle of 60°.

In a similar fashion, we investigate the effect of the *b*-value, on the accuracy of the reconstruction techniques under different SNRs. From the previous discussion, we concluded that the number of 70 gradient direction is sufficient for good detection of a 60° crossing fiber configuration, and therefore in this analysis we fix the number of gradients to 72 (Figure 4). For data with SNR 10, there is a clear tendency for an optimum *b*-value of around 2000 s/mm^2^ as seen in the decrease in the angular error. Higher *b*-values, produce worse results with high standard deviations. However, if the SNRs are higher, the angular error and the standard deviation decay and stabilize around *b*-values between 2000 s/mm^2^ and 3000 s/mm^2^. Again, the results coincide with the ones from the analysis of analytical DOT-ODF, and for completeness are reported in Figures 8, 9 and 10.

**Figure 5:**
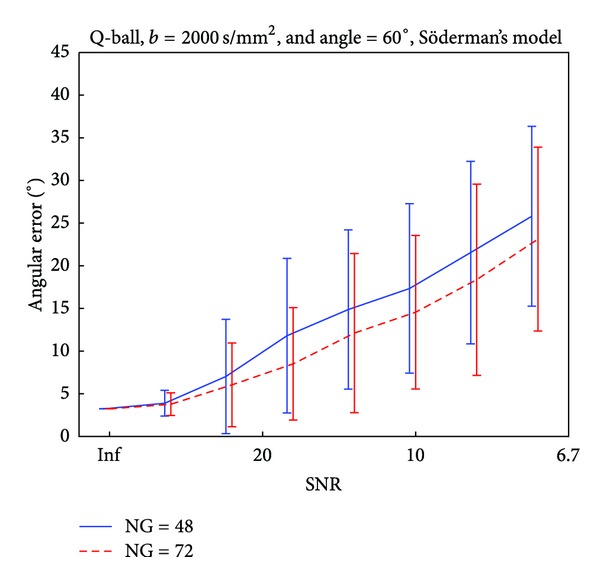
Influence of the SNR on the accuracy of Q-ball imaging for angle of 60°.

The previous analysis emphasizes the importance of the SNRs for reliability in the reconstruction of analytical HARDI decomposition techniques. To address this important issue, we investigate the effect of the SNR under fixed parameters for *b*-value and number of gradients and report in Figure 5. We observe that data with SNRs lower than 15 would produce very unreliable datasets, where the accuracy would drop to an angular error bigger than 10°. Smaller angles cannot be resolved accurately and most probably will not be detected as distinct fiber populations. It can also be seen that the effect of the number of gradient directions (at least at the level between 48 and 72) has much less of an effect on detection performance than the SNR level does. Take note that this is a simplified model of the diffusion process, where only two fiber populations are simulated. The scenario gets more complicated when more fiber populations interfere within a voxel. Increasing the number of gradients does not significantly improve the angular error in the case of SNR of about 20.

### 3.2. Real Data Analysis

For better appreciation of anatomical locations, we present the results by visualizing the recovered maxima, overlaid over an FA-map. To be able to observe the changes in the shape of the glyph profiles, in Figures 11, 12, 13, 14, 15, and 16 we additionally report the min, max normalized glyphs since the normalization enhances the profiles. However, in the noisy areas this considerably deforms the glyphs, and should be interpreted with care. For the color-coding we use standard RGB directional coloring. We present the results from Q-ball in all our figures since they are almost identical with the ones from the DOT-ODF (see Figures 1(a) and 1(e)).

**Figure 6:**
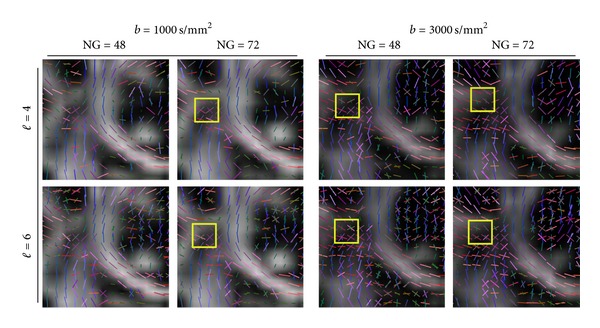
The effect of the truncation order of the spherical harmonics. The yellow rectangle marks the expected three-fold crossing of the CC, CR, and SLF. We observe more three-fold crossings at order *ℓ* = 3. Additionally, *b* = 3000 s/mm^2^ performs better than *b* = 1000 s/mm^2^, since more threefold crossings are recovered.

Figure 6 illustrates the effect of the *ℓ*-order acquired under different parameters. The visualization is by recovered maxima. We observe that in all of the cases, the threefold crossing is better recovered at order 6, at the higher *b*-value (3000 s/mm^2^), and higher number of gradients (72). At these parameter settings the structure of the SLF (yellow rectangle) becomes more evident. Therefore, we conclude that SH order *ℓ* = 6 is the optimal for recovering the crossing information in this region. This statement corroborates our findings from the synthetic data. Going to orders higher than *ℓ* = 6 results in many false maxima detected from the spurious peaks coming from the noise. Additionally, we observe that *b* = 3000 s/mm^2^ performs better than *b* = 1000 s/mm^2^, since more threefold crossings are detected in the *centrum semiovale*. 

The key investigation that answers the question of clinically realizable HARDI protocols is of the effect the *b*-value (and thus the SNR level) and the number of gradients on the accuracy of the analytical decomposition techniques like Q-ball and DOT (and its previously defined derivations DOT-ODF and DOT-mODF). From the previous synthetic analysis, we observed that at about a number of 70 gradients the angular error stabilizes and does not decrease significantly when increasing the number of gradients. However, it is interesting to investigate how low the number of gradients can go at a given *b*-value and SNR level, such that the data still has reasonable reconstructions and detected crossings. Therefore, we select gradient schemes with NG lower than 72 to investigate this question.

**Figure 7:**
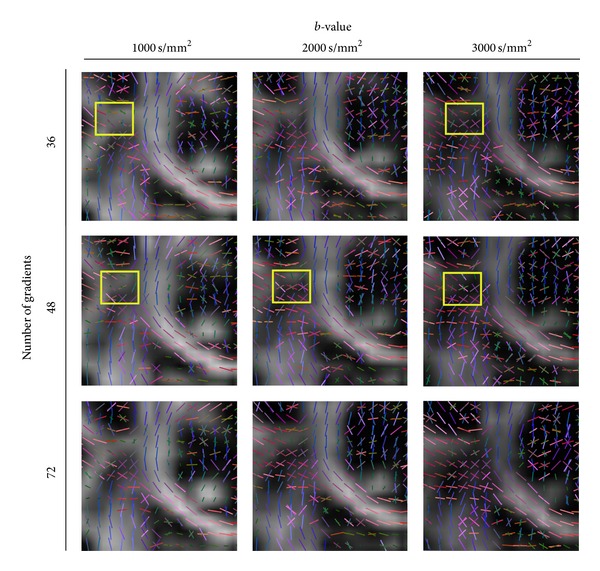
The effect of the *b*-value and the number of gradients at *ℓ* = 6. We observe that the crossings from CC and CR are already observed in the lowest parameter combination of *b* = 1000 s/mm^2^ and NG = 36. The SLF structures become more prominent at higher *b* ≥ 2000 s/mm^2^ (see yellow marked rectangles).

**Figure 8:**
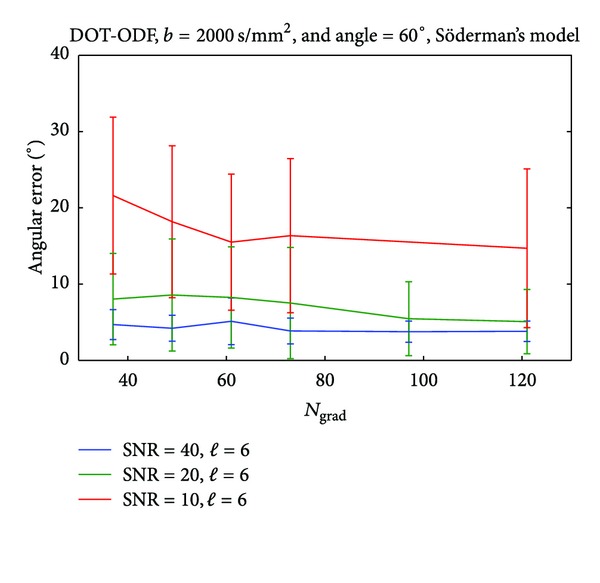
Influence of the gradient sampling schemes on the accuracy of the analytical DOT-ODF imaging for angle of 60°.

**Figure 9:**
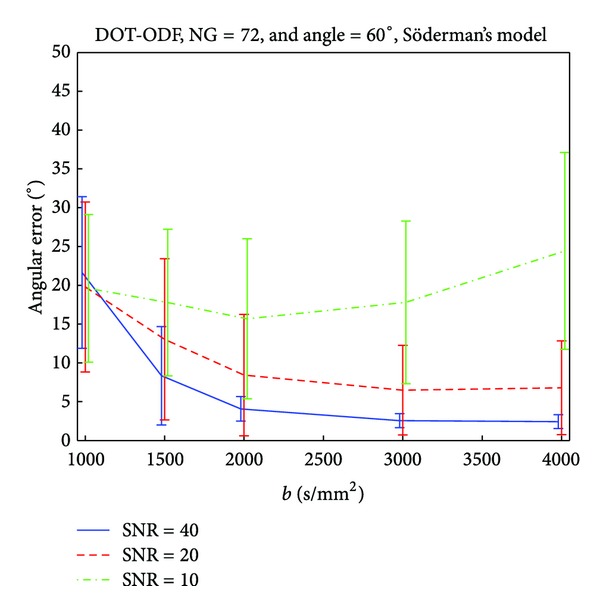
Influence of the *b*-value on the accuracy of the analytical DOT-ODF imaging for angle of 60°.

**Figure 10:**
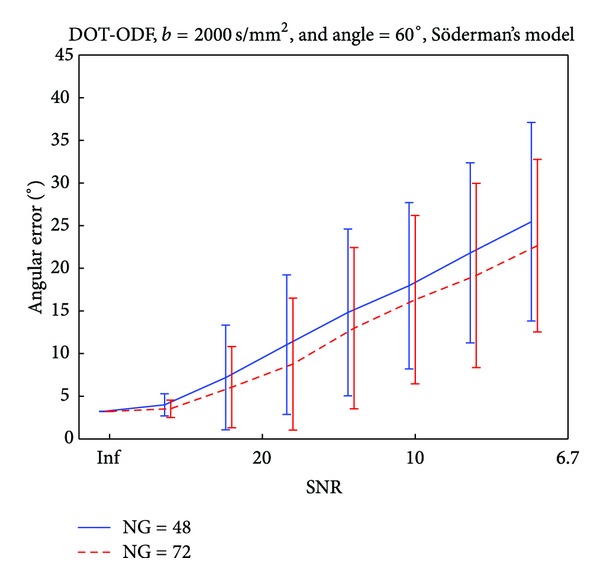
Influence of the SNR on the accuracy of the analytical DOT-ODF imaging for angle of 60°.

**Figure 11:**
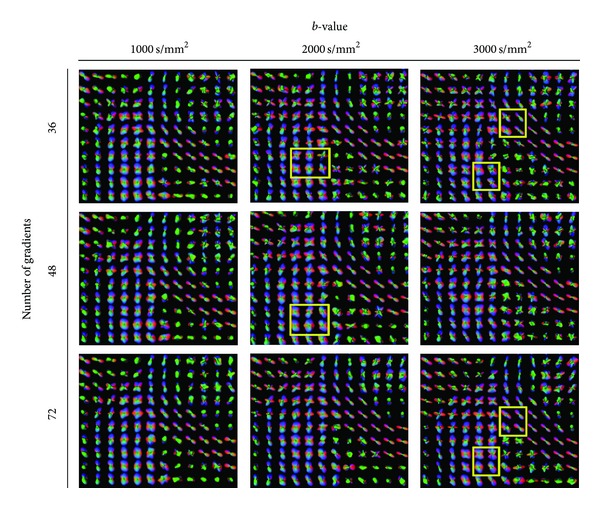
The effect of the *b*-value and the number of gradients at *l* = 6. At higher *b*-values we observe many spurious peaks that disappear with the increase of the number of gradients (see yellow marked rectangles). At NG ≥ 46 there is not a significant difference between the profiles at *b* = 2000 s/mm^2^ and *b* = 3000 s/mm^2^. Given that the spurious peaks are slightly more prominent at *b* = 3000 s/mm^2^, and that lower *b*-values are more attractive with respect to the total scanning time, we conclude that *b* = 2000 s/mm^2^ and NG ∈ [48; 72] are the optimal combination of parameters.

**Figure 12:**
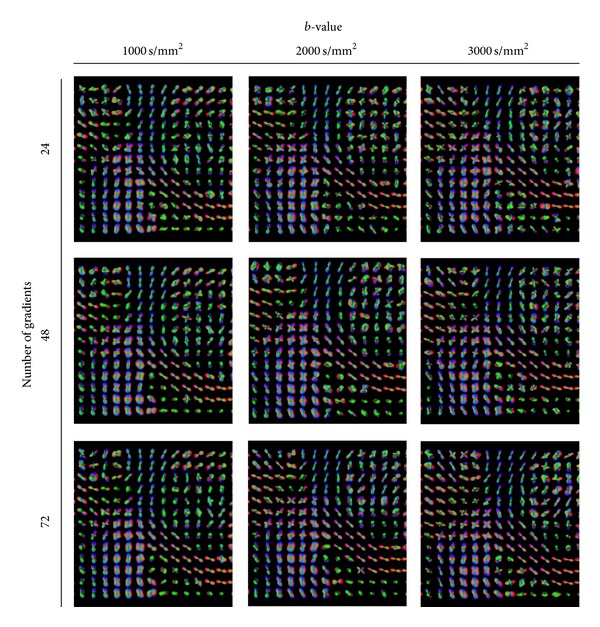
The effect of the *b*-value and the number of gradients at *l* = 4. Conclusions are similar to Figure 11, however, at order *ℓ* = 4 it is more difficult to spot the threefold crossings.

**Figure 13:**
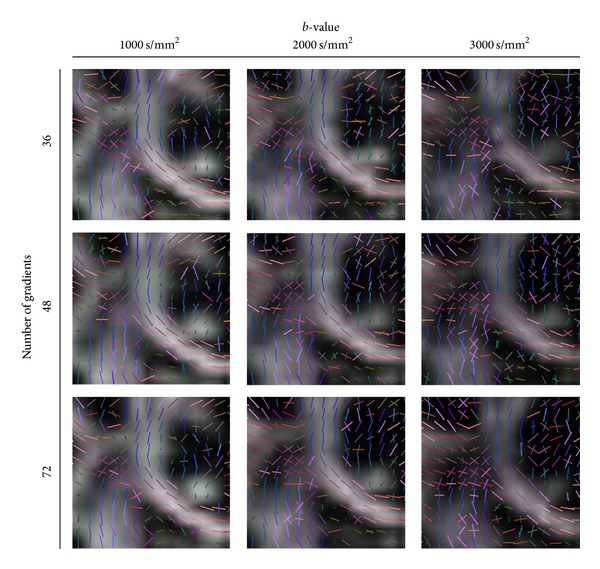
The effect of the *b*-value and the number of gradients at *l* = 4, maxima representation. Conclusions are the same as in Figure 12.

**Figure 14:**
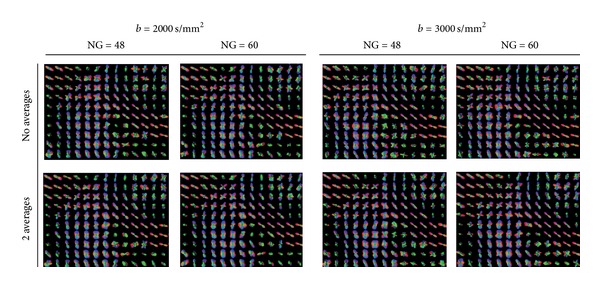
The effect of the averaging of the data at *l* = 6 in a Q-ball glyph representation. There is not a significant difference between the quality of the data without and with averaging even at higher *b*-values.

**Figure 15:**
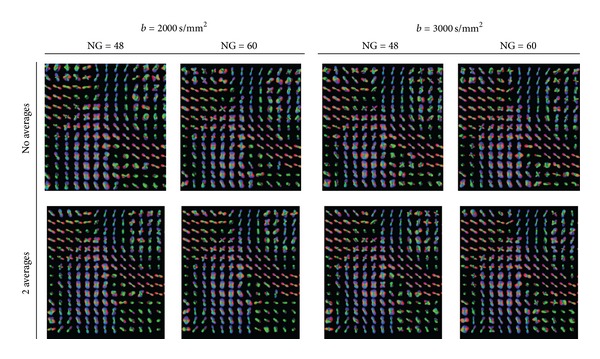
The effect of the averaging of the data at *l* = 4 in a Q-ball glyph representation. There is not a significant difference between the quality of the data without and with averaging even at higher *b*-values.

**Figure 16:**
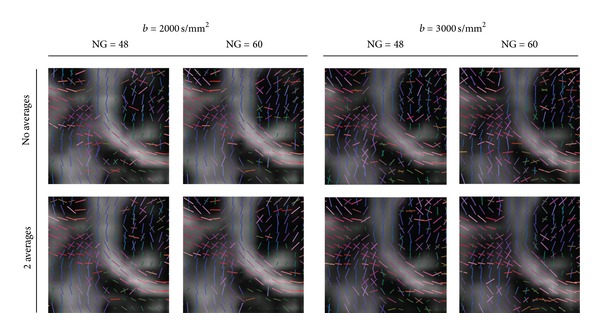
The effect of the averaging of the data at *l* = 4 represented by the maxima detected from Q-ball glyphs. There is not a significant difference between the quality of the data without and with averaging even at higher *b*-values.

In Figure 7, we observe this influence of the *b*-value and NG (for different *ℓ*-orders and glyph representations refer to Figures 11, 12, and 13). We observe that the crossings from CC and CR are already observed in the lowest parameter combination of *b* = 1000 s/mm^2^ and NG = 36 (for order *ℓ* = 4 already at NG = 24). However, the SLF structures are not that obvious and become more prominent at higher *b* ≥ 2000 s/mm^2^ (see yellow marked rectangles in Figure 7). However at higher *b*-values we observe many spurious peaks that disappear with the increase of the number of gradients (this can be better observed in Figure 11). At NG ≥ 48 there is no appreciable difference between the profiles at *b* = 2000 s/mm^2^ and *b* = 3000 s/mm^2^. Given that the spurious peaks are slightly more prominent at *b* = 3000 s/mm^2^, and that lower *b*-values are more attractive with respect to the total scanning time, we conclude that *b* = 2000 s/mm^2^ and NG ∈ [48 − 72] would be preferable with a total scanning time of about 7–10 minutes. For datasets with SNR of about 20, the accuracy of the reconstructed profiles at angles of about 60° would be with angular error of about 8°.

## 4. Discussion

The benefits from applying HARDI modeling techniques that require modest *q*-space single shell sampling over the classical DTI model are well known. However, once the potentials are clear from the theoretical point of view, the questions about the employment of these techniques in every day practice (clinical or cognitive neuroscience research) arise. In the case of HARDI, not only the modeling techniques are complex, but there are many parameters from the acquisition to the postprocessing of the data that influence the accuracy in the estimation of the underlying diffusion profile. The main bottleneck for applying HARDI in clinical practice or as an added protocol in fMRI investigations is the acquisition time, given that decreasing it generally decreases the signal-to-noise ratio in the data thus inferring the accuracy in the reconstructions. The main parameters that influence the total acquisition time are the number of gradients, the spatial resolution (especially the number of slices), and the number of averages, many of which inevitably influence the SNR of the acquired data (please refer to Appendix B). In this study, we compared only analytical decomposition techniques such as Q-ball and DOT. Recent techniques with single *q*-space shell requirements that are known to have better angular resolution than Q-ball such as the solid angle consideration, Q-ball [27] and spherical deconvolution techniques [11, 28] could be used as well. However, we would expect the relative conclusion regarding empirically optimal short-time protocols to also hold for these techniques, possibly at a higher overall accuracy level.

Finding the optimal parameters for the acquisition schemes in terms of both the direction sets and *b*-value has been an open issue in HARDI acquisitions and a few investigations have partially tackled this issue. Jones [29] investigated the effect of gradient sampling schemes on the correctness of estimating the diffusion tensor (DT) and scalar indices derived from it. However, this study was limited to DTI where the effect of the *b*-value is not as significant as in HARDI-based modeling techniques, and therefore *b*-values were not investigated. Moreover, it is based purely on computer simulated data. Alexander and Barker [30] investigated the effect of the *b*-value on a correct estimation of fiber orientations. This Monte Carlo study was limited only to functions that are a mixture of Gaussian distributions and a reconstruction of algorithms that fit this same class or models to synthetic data. There have been a few recent studies that investigated the effect of the number of gradient direction on real experimental data. Zhan et al. [31] focused on a user study for investigating the effect of the number of gradients on the signal-to-noise ratio (SNR) of DTI derived scalar indices. Tournier et al. [32] focused on finding the minimum required number of gradients for successfully reconstructing HARDI diffusion profiles. Other studies have used a hardware diffusion phantom to investigate the effect of measurement parameters on the accuracy of directional diffusion estimation. Cho et al. [33] concentrated on finding the optimal *b*-value for resolving small angles of crossing (in this case 45°) using Q-ball imaging. In Poupon et al. [34] new hardware diffusion phantoms are proposed to similarly investigate the implications on the optimal *b*-value for reconstructing 45° of crossing with Q-ball imaging. Tournier et al. [35] report an extensive study on hardware phantoms comparing the angular resolutions of Q-ball Imaging, Spherical Deconvolution [36], and constrained spherical deconvolution (CSD) [11]. However, in this study the effect of gradient sampling was not examined. Crucially, all of these studies focussed on a situation where acquisition time constraints were very lenient (long diffusion-only protocols on experienced subjects) or absent (phantom and simulation studies). This allowed them to perform their investigation of optimal sampling schemes in a very favorable SNR regime.

In this work, we investigated the relations between above mentioned parameters. We addressed some important questions that relate the SNR of the acquired data with the accuracy of the reconstructions of some popular HARDI analytical decomposition techniques: Q-ball and DOT. To make both of the techniques comparable, we derived from the DOT two numerical ODFs: the DOT-ODF (comparable to the ODF derived from Q-ball) and the DOT-mODF. To utilize the performance of the DOT-ODF, we derive an analytical solution that is comparable to the speed of the DOT and Q-ball. Finally, with synthetic data simulation, and series of real data acquisitions, we gave implications that recommend a set of optimal acquisition parameters for these techniques. If the data is represented by *ℓ* = 6 order of spherical harmonics, an empirically optimal combination (given stringent time constraints) of *b*-value and number of gradients that would resolve most of the angles ≥60° with error of less than 10° is *b* = 2000 s/mm^2^ and NG = 72. However, even at a more modest combination of the acquisition parameters (*b* = 1000 s/mm^2^ and NG = 24) most of the two fiber populations of larger angles will be resolved under the cost of larger angular error. Moreover, these parameters are recommended, given that the data has a minimal SNR (in the 0-weighted acquisitions) of about 20. Decreasing the SNR (increasing the spatial resolution or increasing acquisition speed by techniques such as parallel imaging) would produce unreliable results with angular errors of more than 30°.

It is important to remark that these conclusions to a very large degree hold even for radically different MRI hardware (e.g., high performance head gradient sets, phased-array RF coils with ≥32 channels, or even higher main field strengths than 3T) or new acquisition developments (e.g., highly accelerated parallel imaging) when two observations are taken into account. First, many techniques that are able to speed up the acquisition of a given protocol (e.g., parallel imaging) have an inevitable cost in the SNR level (see Appendix B). Therefore, employing them does not change the fundamental trade-off between spatial resolution, acquisition time, and SNR that define the playing field for the empirical optimization presented here. Second, where the available acquisition technology allows a fundamentally higher SNR or shorter acquisition times (e.g. phased-array coils, simultaneous multi-slice or multi-band acquisitions, and higher field strengths), it stands to reason that this margin would be used to increase the *spatial* resolution of the acquisition beyond the 2.5 mm or 2 mm isotropic voxel level, given that a satisfactory angular resolution and total acquisition time has been obtained. Particularly, when the final processing aim of a study is fiber tractography, there is much to gain in the veridicality of tracked fiber bundles by decreasing partial volume effects and increasing the ability to resolve finer white matter structures (see, e.g., the work of Roebroeck et al. [37]).

## Supplementary Material

Table 1: Results per *b*-value for different SNR, using Q-ball, Södermans model, with N_grad_=97, angle=60v and *l*=6. *fix* is the mean angular error,*s*is the standard deviation of the angular error and *p* is the fraction of samples for which we could retrieve two fiber populations. Table 2: Results per *b*-value for different SNR, using analytical DOT ODF, Södermans model, with N_grad_=97, angle=60v and *l*=6. *fix* is the mean angular error, *s*is the standard deviation of the angular error and *p* is the fraction of samples for which we could retrieve two fiber populations. Table 3: Results per *b*-value for different SNR, using DOT, Södermans model, with N_grad_=97, angle=60vand *l*=6. *fix* is the mean angular error *s* is the standard deviation of the angular error and *p* is the fraction of samples for which we could retrieve two fiber populations.

## Figures and Tables

**Table 1:** Direction tables from our data.

# gradient directions	24	36	48	60	72	96	120
# zero-weighted	3	4	5	6	7	9	11

total # acquisitions	27	40	53	66	79	105	131

**Table 2:** Parameters for the synthetic data similar to the one from our real data measurements.

Parameters					
*b* (s/mm^2^)	1000	1500	2000	3000	4000
Δ (ms)	32.44	36.18	39.18	43.58	47.18
*δ* (ms)	25.34	29.04	32.08	36.48	40.08
*t* (ms)	24.0	26.5	28.5	31.4	33.8

**Table 3:** Approximate whole brain measurement times for a single repetition in (min:sec) with the acquired protocol.

*b*-value	24 dirs	36 dirs	48 dirs	60 dirs	72 dirs
1000	2:58	4:24	5:50	7:16	8:41
1500	3:14	4:48	6:22	7:55	9:29
2000	3:31	5:12	6:53	8:35	10:16
3000	3:47	5:36	7:25	9:14	11:04

**Table 4:** Söderman's model without added noise, with tolerance: 0.5°. The table presents the optimal combination of *b*-value (s/mm^2^) and *l*-order together with the detected angular error (°) for each combination of simulated angle and number of gradients.

Tag	*N*_grad_	40°	45°	50°	55°	60°	90°
	24	No crossing!	No crossing!	No crossing!	No crossing!	10.6 *b* = 3000, *l* = 4	0.1 *b* = 1000, *l* = 4
	36	No crossing!	5.2 *b* = 4000, *l* = 6	1.3 *b* = 4000, *l* = 6	0.1 *b* = 4000, *l* = 6	1.1 *b* = 3000, *l* = 6	0.1 *b* = 1000, *l* = 4
	48	3.9 *b* = 4000, *l* = 8	2.3 *b* = 4000, *l* = 8	1.6 *b* = 4000, *l* = 6	1.1 *b* = 4000, *l* = 6	0.6 *b* = 4000, *l* = 6	0.1 *b* = 1000, *l* = 4
Q-ball	60	4.6 *b* = 4000, *l* = 8	1.9 *b* = 4000, *l* = 8	1.4 *b* = 4000, *l* = 6	0.1 *b* = 4000, *l* = 6	1.6 *b* = 3000, *l* = 6	0.1 *b* = 1500, *l* = 4
	72	4.1 *b* = 4000, *l* = 8	1.9 *b* = 4000, *l* = 8	1.4 *b* = 4000, *l* = 6	0.1 *b* = 4000, *l* = 6	0.1 *b* = 4000, *l* = 6	0.1 *b* = 1000, *l* = 4
	96	4.6 *b* = 4000, *l* = 8	1.9 *b* = 4000, *l* = 8	1.4 *b* = 4000, *l* = 6	0.6 *b* = 4000, *l* = 6	0.1 *b* = 4000, *l* = 6	0.6 *b* = 3000, *l* = 4
	120	4.1 *b* = 4000, *l* = 8	1.9 *b* = 4000, *l* = 8	1.4 *b* = 4000, *l* = 6	0.1 *b* = 4000, *l* = 6	0.1 *b* = 4000, *l* = 6	0.6 *b* = 1000, *l* = 4

	24	No crossing!	No crossing!	No crossing!	No crossing!	4.9 *b* = 4000, *l* = 4	0.1 *b* = 1000, *l* = 4
	36	No crossing!	3.2 *b* = 4000, *l* = 6	1.0 *b* = 4000, *l* = 6	0.1 *b* = 4000, *l* = 6	0.6 *b* = 3000, *l* = 6	0.1 *b* = 1000, *l* = 4
	48	3.4 *b* = 4000, *l* = 8	1.6 *b* = 4000, *l* = 8	1.1 *b* = 4000, *l* = 6	0.1 *b* = 4000, *l* = 6	0.1 *b* = 3000, *l* = 6	0.1 *b* = 1000, *l* = 4
DOT-ODF	60	3.6 *b* = 4000, *l* = 8	1.4 *b* = 4000, *l* = 8	1.1 *b* = 4000, *l* = 6	0.1 *b* = 4000, *l* = 6	1.3 *b* = 3000, *l* = 6	0.1 *b* = 1500, *l* = 4
	72	4.1 *b* = 4000, *l* = 8	1.9 *b* = 4000, *l* = 8	1.1 *b* = 4000, *l* = 6	0.1 *b* = 4000, *l* = 6	0.1 *b* = 3000, *l* = 6	0.1 *b* = 1000, *l* = 4
	96	4.1 *b* = 4000, *l* = 8	1.9 *b* = 4000, *l* = 8	1.1 *b* = 4000, *l* = 6	0.1 *b* = 4000, *l* = 6	0.1 *b* = 3000, *l* = 6	0.6 *b* = 2000, *l* = 4
	120	4.1 *b* = 4000, *l* = 8	1.9 *b* = 4000, *l* = 8	1.1 *b* = 4000, *l* = 6	0.6 *b* = 4000, *l* = 6	0.1 *b* = 3000, *l* = 6	0.6 *b* = 1500, *l* = 4

**Table 5:** 

*ℓ*	0	2	4	6	8
${\tilde{I}}_{\ell }$	1/8π	1/16*π*	3/64*π*	5/128*π*	35/1024π

**Table 6:** The SNR relative to that for a TE equal to *T*2 (assumed to be 70 ms) for different *b*-values and two maximum achievable gradient strengths *G*_max⁡_.

	*b*-value (s/mm^2^)	*TE*_min⁡_ (ms) (2∗d + 25)	Rel-SNR *T*2 = 70 ms
*G*_max⁡_ = 35 mT/m	1000	72.0	1.15
	2000	85.2	0.99
	3000	94.6	0.88
	4000	102.2	0.81

*G*_max⁡_ = 60 mT/m	1000	56.6	1.39
	2000	65.8	1.24
	3000	72.4	1.15
	4000	77.6	1.08

## Appendices

### A. Derivation of Analytical DOT-ODF

#### A.1. Diffusion Orientation Transform

The diffusion probability function *p*(**r**) is related to the measured MR diffusion signal *E*(**q**) by a Fourier integral as noted in (1). In the work of Özarslan et al. [10], the point-wise expansion of the plane wave in spherical coordinates is used in order to obtain the following expression:

(A.1) $$\begin{array}{c} p\left(R_{0}u\right)=\sum_{\ell ,m}{\left(-i\right)}^{\ell }Y_{\ell m}\left(u\right)\int Y_{\ell m}{\left(g\right)}^{*}I_{\ell }\left(g,R_{0}\right)dg, \end{array}$$

where

(A.2) $$\begin{array}{c} I_{\ell }\left(g,R_{0}\right)=4\pi \int_{0}^{\infty }q^{2}j_{\ell }\left(2\pi qR_{0}\right)e^{-4\pi^{2}q^{2}tD(g)}dq, \end{array}$$

where *j*_*ℓ*_(2*πqR*_0_) is the *ℓ*th order spherical Besel function. Normally, the radius *R*_0_ is fixed, but one needs to find an optimal radius in order to have a sharp enough profile. In order to avoid this problem, we consider ODF, described in the following section.

#### A.2. ODF from DOT

In order to obtain the ODF, we need to integrate (2):

(A.3) $$\begin{array}{c} \psi \left(u\right)=\sum_{\ell ,m}{\left(-i\right)}^{\ell }Y_{\ell m}\left(u\right)\int Y_{\ell m}{\left(g\right)}^{*}\left(\int_{0}^{\infty }I_{\ell }\left(g,R_{0}\right)dR_{0}\right)dg. \end{array}$$

According to the work of Özarslan et al. [10], *I*_*ℓ*_(**g**, *R*_0_), given by (A.2), can be computed analytically

(A.4) Iℓ(g,R0)=R0ℓΓ((ℓ+3)/2)2ℓ+3π3/2(D(g)t)(ℓ+3)/2Γ((ℓ+3)/2)×1F1(ℓ+32;ℓ+32;−R024D(g)t),

where _1_ *F*_1_ is the confluent hypergeometric function of the first kind.

In order to compute ∫_0_^*∞*^ *I*_*ℓ*_(**g**, *R*_0_)*dR*_0_, we use the integral representation of _1_ *F*_1_,

(A.5) $$\begin{array}{c} {\;}_{1}F_{1}\left(a,b,z\right)=\int_{0}^{1}\xi^{a-1}{\left(1-\xi \right)}^{b-a-1}e^{z\xi }d\xi , \end{array}$$

and change the integration order. As a result, we obtain an analytical formula for the integral

(A.6) $$\begin{array}{c} \int_{0}^{\infty }I_{\ell }\left(g,R_{0}\right)dR_{0}=\frac{1}{D\left(g\right)t}{\tilde{I}}_{\ell }, \end{array}$$

where

(A.7) $$\begin{array}{c} {\tilde{I}}_{\ell }=\frac{\Gamma \left(\left(\ell +1\right)/2\right)}{4\ell \pi^{3/2}\Gamma \left(\ell /2\right)}. \end{array}$$

The values for *ℓ* up to 8 are summarized in Table 5.

Therefore, if 1/*D*(**g**)*t* is given in spherical harmonic representation

(A.8) $$\begin{array}{c} \frac{1}{D\left(g\right)t}=\sum_{\ell ,m}c_{\ell m}Y_{\ell m}\left(g\right), \end{array}$$

(A.3) and (A.6) give us

(A.9) $$\begin{array}{c} \psi \left(u\right)=\sum_{\ell ,m}{\left(-1\right)}^{\ell /2}{\tilde{I}}_{\ell }c_{\ell m}Y_{\ell m}\left(u\right), \end{array}$$

Since tensor terms of order *k* belong to the span of the spherical harmonics of the same order, we conclude that the same holds for the tensor representation

(A.10) $$\begin{array}{c} \psi \left(u\right)=\sum_{\ell ,m}{\left(-1\right)}^{\ell /2}{\tilde{I}}_{\ell }D^{i_{\ell }\cdots i_{k}}u_{i_{1}}\cdots u_{i_{k}}, \end{array}$$

where *D* ^*i*_*ℓ*_⋯*i*_*k*_^-coefficients of tensor expansion for 1/*D*(**g**)*t*.

### B. The Basics of Signal and Noise in Pulse Gradient Spin echo–echo Planar Imaging (PGSE–EPI) Diffusion MR Sequences

Leaving static magnetic field **B0** outside of consideration, the signal for a spin echo experiment is directly proportional to the available transverse magnetization, which is itself a function of TR, T1, TE, and T2:

(B.1) $$\begin{array}{c} M_{⊥}\approx \left(1-e^{-\text{TR}/\text{T}1}\right)e^{-\text{TE}/\text{T}2}. \end{array}$$

To maximize the available signal, TR must be maximized and TE minimized. Given the usual requirement in diffusion MR imaging for whole brain coverage using a 2D (slice selective) sequence, the TR is naturally long, usually longer than 4 times T1 (which is about 800 ms for white matter at 3T), recovering at least 98% of the available signal. In contrast, the achievable TE in a Stejskal-Tanner PGSE sequences is typically long compared to T2 (which is about 84 ms for white matter at 3T), due to the diffusion gradient lobes. The amount of diffusion weighting, when gradient ramps are a small proportion of the diffusion-gradient lobes, is given by the *b*-value:

(B.2) $$\begin{array}{c} b=\gamma^{2}\delta^{2}\left(\Delta -\frac{\delta }{3}\right){\left|G\right|}^{2}, \end{array}$$

where *γ* is the 1H gyromagnetic ratio and |**G**|, Δ, and *δ* are the amplitude, time between onsets, and length of the two diffusion gradients, respectively. Since the *b*-value has a cubic dependence on *δ*, quadratic on |**G**|, and linear on Δ, the most SNR-efficient way (i.e., minimum TE) to achieve any given *b*-value is to play out diffusion gradients at maximum available amplitude |**G**| and keep the Δ as short as possible. Therefore, the SNR in diffusion imaging necessarily falls with increasing *b*-value, through increasing TE. An estimate of the relative SNR for different *b*-values can be made if one estimates a minimum achievable TE as 2∗*δ* + 25 (assuming Δ = *δ* + 5, and 25 ms total for RF pulse lengths, pre-TE readout train length, and additional encoding and spoiling). The SNR relative to that for a TE equal to T2 (assumed to be 70 ms) for different *b*-values and two maximum achievable gradient strengths *G*_max⁡_ is given in the Table 6.

In addition, the SNR for each voxel is crucially dependent on the parameters of the imaging gradients and the readout train in EPI. The SNR in 2D Fourier-encoded MRI is proportional to

(B.3) $$\begin{array}{c} \text{SNR}\propto \Delta x\Delta yTh\sqrt{N_{\text{avg}}N_{y}T_{s}} \end{array}$$

with *T*_*s*_ = *N*_*x*_/*BW*_read_, Δ*x* and Δ*y* are voxel sizes along readout and phase-encoded direction, respectively, *Th* is slice thickness, *N*_avg_ is the number of averaged repetitions of the entire sequence, *N*_*x*_ and *N*_*y*_ are the number of samples along readout and phase-encoded direction, respectively, *T*_*s*_ is the readout sampling time, and *BW*_read_ is the readout bandwidth. This highlights the trade off between SNR, spatial resolution, spatial distortion, and dropout and total imaging time. In particular, for PGSE-EPI sequences, SNR can be traded off in the following ways.

- Increasing voxel size Δ*x* × Δ*y* × *Th* increases the SNR at the cost of spatial resolution and increased susceptibility related distortions and dropout at air-tissue boundaries. Increasing voxel size from [2 × 2 × 2] mm^3^ to [2.5 × 2.5 × 2.5] mm^3^ or [3 × 3 × 3] mm^3^ increases SNR with a factor 1.95 and 3.37, respectively (if other parameters, particularly *N*_*y*_ and *T*_*s*_ are held constant).
- Increasing the number of averaged repetitions of the entire sequence increases SNR at the cost of increased imaging time. Acquiring 2, 3, or 4 averages increases SNR with a factor 1.41, 1.73, and 2, respectively (if the patient does not move between acquisitions).
- Decreasing readout bandwidth increases the SNR at the cost of spatial resolution (due to T2*-decay-related blurring) and increased susceptibility related distortions and dropout. In principle, however, the SNR gain can be partially compensated by increased TE with a longer readout train.
- Increasing the number of phase-encoding steps (equivalently: the number of readouts) increases the SNR when all else is held constant. However, this increases the readout train length and TE, which can compensate the SNR gain. In practice, *N*_*y*_ is almost routinely decreased, by employing either partial Fourier sampling or accelerated (parallel) imaging along the phase encoded direction (or both), allowing a decrease in TE. The shorter readout trains also decrease susceptibility related distortions along the phase-encoded direction. In addition to the factor *N*_*y*_, parallel imaging further decreases the SNR by a spatially varying factor *g*(*x*, *y*) which depends on the coil array geometry and acceleration factor used.

Finally, SNR crucially depends on the type and performance of the employed coil array for RF signal reception. A phased-array coil design can give considerable SNR advantages over a single circularly polarized birdcage design. However, the signal sensitivity for a phased array decreases quadratically with distance from the coil array, towards the center of the head, thus showing considerable spatial inhomogeneity of the SNR. A phased array of 8 coils can have a typical SNR advantage of 100% (6 dB) at the peripheral levels of the brain (gray matter) compared to a birdcage coil of similar dimensions but only 10% more signal-to-noise in the central white matter. Finally, a phased array coil will enable parallel imaging, with the additional SNR trade-off discussed above.
