# Supplementary material for: Optimal Short-Time Acquisition Schemes in High Angular Resolution Diffusion-Weighted Imaging
Source: Int J Biomed Imaging. 2013 Mar 11;2013:658583. doi: 10.1155/2013/658583 (PMC3608261; doi:10.1155/2013/658583)
Supplement: Supplementary file 1 — Table 1: Results per b-value for different SNR, using Q-ball, Södermans model, with Ngrad=97, angle=60v and l=6. fix is the mean angular error,sis the standard deviation of the angular error and p is the fraction of samples for which we could retrieve two fiber populations. Table 2: Results per b-value for different SNR, using analytical DOT ODF, Södermans model, with Ngrad=97, angle=60v and l=6. fix is the mean angular error, sis the standard deviation of the angular error and p is the fraction of samples for which we could retrieve two fiber populations. Table 3: Results per b-value for different SNR, using DOT, Södermans model, with Ngrad=97, angle=60vand l=6. fix is the mean angular error s is the standard deviation of the angular error and p is the fraction of samples for which we could retrieve two fiber populations. [file 658583.f1.pdf]

Tables for optimal b-value selection

	Signal to Noise Ratio (SNR)							
**b-value** (s/mm^2)	∞	50.0	25.0	16.7	12.5	10.0	8.3	5.0
**1000**	$\bar{x}$: 60.0	$\bar{x}$: 11.0	$\bar{x}$: 9.3	$\bar{x}$: 14.1	$\bar{x}$: 16.9	$\bar{x}$: 18.0	$\bar{x}$: 19.4	$\bar{x}$: 24.8
	s 0.0:	s 2.5:	s 4.7:	s 9.8:	s 10.1:	s 10.4:	s 9.6:	s 9.9:
	p: 0.00	p: 0.46	p: 0.44	p: 0.62	p: 0.82	p: 0.93	p: 0.91	p: 0.99
**1500**	$\bar{x}$: 5.7	$\bar{x}$: 6.3	$\bar{x}$: 6.4	$\bar{x}$: 10.0	$\bar{x}$: 12.3	$\bar{x}$: 12.8	$\bar{x}$: 16.0	$\bar{x}$: 27.2
	s 0.0:	s 1.9:	s 2.8:	s 9.3:	s 9.7:	s 8.9:	s 9.5:	s 8.9:
	p: 1.00	p: 0.99	p: 0.92	p: 0.83	p: 0.96	p: 0.95	p: 1.00	p: 0.98
**2000**	$\bar{x}$: 3.2	$\bar{x}$: 3.4	$\bar{x}$: 4.6	$\bar{x}$: 7.1	$\bar{x}$: 10.9	$\bar{x}$: 15.4	$\bar{x}$: 16.1	$\bar{x}$: 26.5
	s 0.0:	s 1.1:	s 3.2:	s 6.9:	s 8.9:	s 10.9:	s 9.3:	s 10.7:
	p: 1.00	p: 1.00	p: 0.98	p: 0.96	p: 0.95	p: 0.95	p: 1.00	p: 0.98
**3000**	$\bar{x}$: 1.1	$\bar{x}$: 1.9	$\bar{x}$: 3.1	$\bar{x}$: 5.7	$\bar{x}$: 8.9	$\bar{x}$: 14.5	$\bar{x}$: 19.1	$\bar{x}$: 30.8
	s 0.0:	s 0.7:	s 1.2:	s 3.9:	s 6.6:	s 9.6:	s 10.0:	s 10.6:
	p: 1.00	p: 1.00	p: 1.00	p: 0.99	p: 0.99	p: 1.00	p: 0.99	p: 0.89
**4000**	$\bar{x}$: 0.1	$\bar{x}$: 1.4	$\bar{x}$: 3.1	$\bar{x}$: 6.1	$\bar{x}$: 12.0	$\bar{x}$: 19.2	$\bar{x}$: 23.8	$\bar{x}$: 32.5
	s 0.0:	s 0.6:	s 1.5:	s 5.0:	s 9.1:	s 11.8:	s 11.1:	s 11.2:
	p: 1.00	p: 1.00	p: 1.00	p: 1.00	p: 1.00	p: 0.97	p: 0.99	p: 0.88

Table 1

Results per b-value for different SNR, using Q-ball, Södermans model, with N_{grad}=97, angle=60°and ℓ=6. $\bar{x}$ is the mean angular error, s is the standard deviation of the angular error and p is the fraction of samples for which we could retrieve two fiber populations.

	Signal to Noise Ratio (SNR)							
**b-value** (s/mm^2)	∞	50.0	25.0	16.7	12.5	10.0	8.3	5.0
**1000**	$\bar{x}$: 60.0	$\bar{x}$: 12.2	$\bar{x}$: 16.2	$\bar{x}$: 16.2	$\bar{x}$: 19.6	$\bar{x}$: 18.9	$\bar{x}$: 19.0	$\bar{x}$: 25.9
	s 0.0:	s 5.5:	s 10.4:	s 11.3:	s 10.9:	s 10.0:	s 9.7:	s 10.3:
	p: 0.00	p: 0.16	p: 0.55	p: 0.75	p: 0.85	p: 0.94	p: 0.96	p: 0.95
**1500**	$\bar{x}$: 6.2	$\bar{x}$: 6.5	$\bar{x}$: 8.2	$\bar{x}$: 10.6	$\bar{x}$: 13.8	$\bar{x}$: 15.1	$\bar{x}$: 17.3	$\bar{x}$: 25.6
	s 0.0:	s 1.7:	s 6.6:	s 9.4:	s 10.9:	s 10.1:	s 10.5:	s 9.9:
	p: 1.00	p: 0.97	p: 0.93	p: 0.88	p: 0.95	p: 0.98	p: 0.98	p: 1.00
**2000**	$\bar{x}$: 3.2	$\bar{x}$: 3.5	$\bar{x}$: 4.8	$\bar{x}$: 9.6	$\bar{x}$: 13.4	$\bar{x}$: 14.9	$\bar{x}$: 16.8	$\bar{x}$: 27.0
	s 0.0:	s 1.1:	s 4.0:	s 9.1:	s 11.0:	s 9.9:	s 10.4:	s 10.4:
	p: 1.00	p: 1.00	p: 0.99	p: 0.98	p: 1.00	p: 0.99	p: 1.00	p: 1.00
**3000**	$\bar{x}$: 0.6	$\bar{x}$: 1.8	$\bar{x}$: 3.3	$\bar{x}$: 5.4	$\bar{x}$: 9.6	$\bar{x}$: 12.3	$\bar{x}$: 20.1	$\bar{x}$: 30.7
	s 0.0:	s 0.6:	s 1.5:	s 4.1:	s 8.3:	s 8.6:	s 11.8:	s 11.6:
	p: 1.00	p: 1.00	p: 1.00	p: 1.00	p: 0.99	p: 1.00	p: 0.99	p: 0.95
**4000**	$\bar{x}$: 0.1	$\bar{x}$: 1.6	$\bar{x}$: 3.2	$\bar{x}$: 7.0	$\bar{x}$: 12.4	$\bar{x}$: 18.3	$\bar{x}$: 23.0	$\bar{x}$: 33.2
	s 0.0:	s 0.6:	s 1.2:	s 6.2:	s 9.5:	s 11.9:	s 11.6:	s 9.1:
	p: 1.00	p: 1.00	p: 1.00	p: 1.00	p: 0.99	p: 1.00	p: 0.99	p: 0.93

Table 2

Results per b-value for different SNR, using analytical DOT ODF, Södermans model, with N_{grad}=97, angle=60°and ℓ=6. $\bar{x}$ is the mean angular error, s is the standard deviation of the angular error and p is the fraction of samples for which we could retrieve two fiber populations.

	Signal to Noise Ratio (SNR)							
**b-value** (s/mm^2)	∞	50.0	25.0	16.7	12.5	10.0	8.3	5.0
**1000**	$\bar{x}$: 1.1	$\bar{x}$: 2.9	$\bar{x}$: 6.3	$\bar{x}$: 11.2	$\bar{x}$: 19.2	$\bar{x}$: 24.6	$\bar{x}$: 28.4	$\bar{x}$: 34.2
	s 0.0:	s 1.4:	s 5.2:	s 9.1:	s 12.1:	s 12.6:	s 10.7:	s 9.3:
	p: 1.00	p: 1.00	p: 0.92	p: 0.90	p: 0.87	p: 0.87	p: 0.84	p: 0.68
**1500**	$\bar{x}$: 0.6	$\bar{x}$: 1.9	$\bar{x}$: 3.4	$\bar{x}$: 5.7	$\bar{x}$: 12.5	$\bar{x}$: 20.0	$\bar{x}$: 24.2	$\bar{x}$: 32.0
	s 0.0:	s 0.8:	s 1.4:	s 3.6:	s 10.2:	s 12.1:	s 11.8:	s 9.2:
	p: 1.00	p: 1.00	p: 0.98	p: 0.95	p: 0.88	p: 0.91	p: 0.90	p: 0.82
**2000**	$\bar{x}$: 0.6	$\bar{x}$: 1.5	$\bar{x}$: 3.0	$\bar{x}$: 5.3	$\bar{x}$: 10.4	$\bar{x}$: 19.2	$\bar{x}$: 26.6	$\bar{x}$: 32.5
	s 0.0:	s 0.6:	s 1.2:	s 3.3:	s 9.3:	s 13.2:	s 13.3:	s 9.7:
	p: 1.00	p: 1.00	p: 1.00	p: 0.98	p: 0.88	p: 0.87	p: 0.95	p: 0.74
**3000**	$\bar{x}$: 1.0	$\bar{x}$: 1.6	$\bar{x}$: 2.8	$\bar{x}$: 6.4	$\bar{x}$: 13.8	$\bar{x}$: 21.5	$\bar{x}$: 28.6	$\bar{x}$: 35.9
	s 0.0:	s 0.6:	s 1.0:	s 6.0:	s 11.2:	s 13.5:	s 11.1:	s 9.7:
	p: 1.00	p: 1.00	p: 1.00	p: 0.97	p: 0.94	p: 0.85	p: 0.83	p: 0.67
**4000**	$\bar{x}$: 0.1	$\bar{x}$: 1.7	$\bar{x}$: 3.4	$\bar{x}$: 8.4	$\bar{x}$: 22.5	$\bar{x}$: 28.9	$\bar{x}$: 31.0	$\bar{x}$: 33.4
	s 0.0:	s 0.7:	s 1.4:	s 9.3:	s 13.1:	s 13.1:	s 10.9:	s 10.3:
	p: 1.00	p: 1.00	p: 1.00	p: 0.95	p: 0.98	p: 0.83	p: 0.80	p: 0.59

Table 3

Results per b-value for different SNR, using DOT, Södermans model, with N_{grad}=97, angle=60°and ℓ=6. $\bar{x}$ is the mean angular error, s is the standard deviation of the angular error and p is the fraction of samples for which we could retrieve two fiber populations.
